# Benign Movement Disorders Mimicking Seizures in Children: A Retrospective Cohort Study

**DOI:** 10.3390/children13050650

**Published:** 2026-05-06

**Authors:** Arzu Eroglu

**Affiliations:** Pediatric Neurology Clinic, Balıkesir Atatürk City Hospital, Balıkesir 10100, Türkiye; mdarzueroglu@hotmail.com

**Keywords:** benign movement disorders, breath-holding spells, Sandifer syndrome, non-epileptic events, children, seizures, EEG, misdiagnosis

## Abstract

**Highlights:**

**What are the main findings?**

- Nearly one in four children referred with suspected seizures had benign movement disorders.
- EEG findings were predominantly normal, and no patients developed epilepsy.

**What are the implications of the main findings?**

- Improved recognition of these conditions may reduce epilepsy misdiagnosis.
- Early diagnosis can help avoid unnecessary investigations and treatment.

**Abstract:**

**Background:** Developmental and benign movement disorders (DBMD) are a common but often under-recognized cause of referral in children with suspected seizures, frequently leading to misdiagnosis and unnecessary treatment. **Methods:** This retrospective cohort study included 453 children evaluated for suspected seizures between January 2019 and January 2024. Patients with epilepsy, cerebral palsy, metabolic disorders, significant developmental delay, psychiatric conditions, or structural brain abnormalities were excluded. DBMD was diagnosed in 113 patients based on clinical evaluation by experienced pediatric neurologists and established diagnostic criteria. Demographic characteristics, diagnostic distribution, age at presentation, and electroencephalography (EEG) findings were analyzed. Prevalence was calculated with 95% confidence intervals (CIs). Group comparisons were performed using chi-square tests and one-way ANOVA with Tukey post hoc analysis. **Results:** The prevalence of DBMD was 24.9% (95% CI: 21.2–29.1), corresponding to nearly one in four children referred with suspected seizures. Breath-holding spells were the most common diagnosis (6.6% of the total cohort; 26.5% of DBMD cases), followed by Sandifer syndrome and non-epileptic staring episodes. Age at presentation differed significantly between diagnostic groups (*p* = 0.001), with breath-holding spells occurring at younger ages and staring episodes at older ages. EEG findings were normal in 80.5% of patients, and no diagnosis-specific epileptiform patterns were identified. No patients were observed to develop epilepsy during the follow-up period. **Conclusions:** DBMD accounts for a substantial proportion of children referred for suspected seizures. Recognition of age-specific clinical patterns and predominantly normal EEG findings may improve diagnostic accuracy and help avoid unnecessary investigations and antiepileptic treatment.

## 1. Introduction

Developmental and benign movement disorders (DBMDs) constitute a significant subgroup of non-epileptic paroxysmal events in childhood and are frequently misdiagnosed as epileptic seizures in clinical practice [1,2]. These conditions typically arise during specific stages of neurodevelopment and are characterized by normal neurological examination, absence of structural brain pathology, and a generally favorable prognosis [1]. The concept of transient benign paroxysmal movement disorders in infancy was systematically described by Fernández-Alvarez, who emphasized their developmental and self-limited nature [2]. Increasing evidence suggests that many of these phenomena reflect age-dependent immaturity of neuronal networks and autonomic regulation rather than true epileptic activity [1,3]. Accordingly, they may represent physiological manifestations of ongoing brain maturation.

Common DBMD entities include breath-holding spells, Sandifer syndrome, benign sleep myoclonus, shuddering attacks, and motor stereotypies [1,4,5]. Breath-holding spells, typically occurring between 6 and 24 months of age, are thought to result from autonomic dysregulation and exaggerated vagal responses [6]. Sandifer syndrome, characterized by dystonic posturing associated with gastroesophageal reflux, can closely mimic focal seizures and often leads to unnecessary neurological investigations and treatment [7]. Similarly, benign sleep myoclonus and shuddering attacks are well-recognized non-epileptic conditions with normal electroencephalographic findings [1,4]. Previous studies indicate that DBMDs account for approximately 20% of childhood movement disorders and represent a substantial proportion of paroxysmal non-epileptic events referred for seizure evaluation [1,2]. However, data specifically evaluating their prevalence in large cohorts of children presenting with suspected seizures remain limited. In addition, comparative analyses examining age-related differences across diagnostic subtypes are scarce. Accurate recognition of DBMDs is essential to prevent epilepsy misdiagnosis, avoid unnecessary antiepileptic treatment, reduce healthcare costs, and alleviate parental anxiety. Improved understanding of their developmental timing and clinical characteristics may enhance diagnostic accuracy in pediatric neurology practice.

The aim of this study was to determine the prevalence, clinical characteristics, and age-related presentation patterns of developmental and benign movement disorders in a large cohort of children referred with suspected seizures in a tertiary pediatric neurology center.

## 2. Materials and Methods

### 2.1. Study Design and Setting

This retrospective cohort study was conducted at the Pediatric Neurology Clinic of Balıkesir Atatürk City Hospital, a tertiary referral center. Medical records of children evaluated for suspected seizures between January 2019 and January 2024 were reviewed.

### 2.2. Patient Population

A total of 453 children aged 0–18 years who were referred with suspected seizures were screened. Patients with confirmed epilepsy, cerebral palsy, metabolic disorders, significant developmental delay, psychiatric disorders, or structural brain abnormalities were excluded. After applying the exclusion criteria, 113 patients were diagnosed with developmental and benign movement disorders (DBMDs).

### 2.3. Diagnostic Evaluation

All patients underwent comprehensive clinical assessment, including detailed medical history and neurological examination performed by experienced pediatric neurologists. Electroencephalography (EEG) recordings were performed using the standard international 10–20 system when clinically indicated. Brain magnetic resonance imaging (MRI), metabolic screening, and additional investigations were obtained when necessary to exclude epileptic, metabolic, or structural etiologies. Clinical video recordings, when available, were systematically reviewed and used as supportive diagnostic tools to differentiate epileptic from non-epileptic events. Clinical video recordings were reviewed in all patients. EEG recordings were performed during both wakefulness and sleep; however, EEG recordings during clinical events and prolonged video-EEG monitoring were not systematically performed. DBMD diagnoses were established based on previously described clinical criteria, normal neurological examination, absence of epileptiform EEG findings, and follow-up data demonstrating a benign clinical course.

### 2.4. Data Collection

The following variables were recorded: age at presentation (months), sex, final clinical diagnosis, EEG findings (normal vs. non-specific abnormalities), and follow-up duration (months). The prevalence of DBMD was calculated relative to the total cohort (n = 453) and within the DBMD subgroup (n = 113).

### 2.5. Statistical Analysis

Statistical analyses were performed using SPSS software (version 25.0, IBM Corp., Armonk, NY, USA). Continuous variables were expressed as mean ± standard deviation (SD), and categorical variables as number and percentage. Prevalence estimates were calculated with 95% confidence intervals (CIs) using the Wilson method. Categorical variables were compared using the chi-square test. Differences in age at presentation among diagnostic groups were analyzed using one-way analysis of variance (ANOVA), followed by Tukey post hoc test for pairwise comparisons. A *p*-value < 0.05 was considered statistically significant.

### 2.6. Ethics Approval

This study was conducted in accordance with the Declaration of Helsinki and approved by the Scientific Research Ethics Committee of Balıkesir Atatürk City Hospital (Decision No: 2014/12/75; Date: 26 December 2024).

### 2.7. Informed Consent

Due to the retrospective design of the study, the requirement for informed consent was waived by the ethics committee. Written informed consent was obtained from the legal guardians for the recording and scientific use of clinical video materials. All video data were anonymized prior to analysis to ensure patient confidentiality.

## 3. Results

### 3.1. Age-Related Differences

Age at presentation differed significantly among diagnostic groups (one-way ANOVA, F = 5.12, *p* = 0.001; Cohen’s f = 0.27), indicating a moderate effect size (Table 1). Post hoc Tukey analysis demonstrated that breath-holding spells occurred at significantly younger ages compared to Sandifer syndrome (mean difference: 5.65 months, *p* = 0.0017). In contrast, non-epileptic staring episodes presented at significantly older ages compared to Sandifer syndrome (mean difference: 5.28 months, *p* = 0.027).

**Figure 1:**
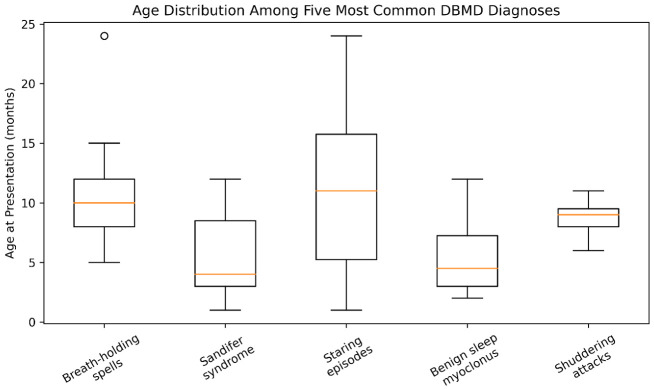
Age distribution among the five most common developmental and benign movement disorders (DBMD). Boxplot analysis demonstrates differences in age at presentation across diagnostic groups. Breath-holding spells tend to occur at younger ages, whereas non-epileptic staring episodes present at older ages. Horizontal lines indicate medians, boxes represent interquartile ranges, and whiskers denote minimum and maximum values excluding outliers.

A post hoc power analysis (effect size f = 0.27, α = 0.05, k = 5 groups, n = 113) demonstrated a statistical power of 0.64, indicating moderate power to detect medium-sized effects. Age distribution across diagnostic subgroups is illustrated in Figure 1. 

### 3.2. Diagnostic Distribution

Breath-holding spells were the most frequent diagnosis (n = 30), representing 6.6% of the total cohort and 26.5% of DBMD cases. Sandifer syndrome was identified in 15 patients (3.3% of the total cohort; 13.3% of DBMD cases), followed by non-epileptic staring episodes in 12 patients (2.6% of the total cohort; 10.6% of DBMD cases).

**Figure 2:**
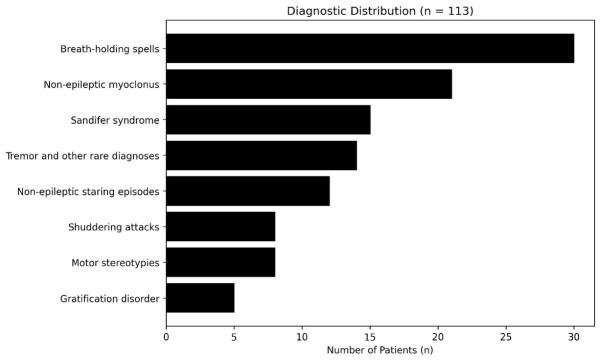
Diagnostic distribution of developmental and benign movement disorders (DBMDs) in children referred with suspected seizures (n = 113). Breath-holding spells constituted the largest diagnostic subgroup (26.5%), followed by Sandifer syndrome (13.3%) and non-epileptic staring episodes (10.6%), highlighting the predominance of autonomic and developmental non-epileptic phenomena within the cohort.

Benign sleep myoclonus accounted for 8.8% of DBMD cases, while shuddering attacks and motor stereotypies each represented 7.1% of the subgroup (Table 2). The distribution of diagnostic categories is illustrated in Figure 2.

### 3.3. EEG Findings and Clinical Outcome

Electroencephalography was normal in 91 patients (80.5%), while non-specific abnormalities were observed in 22 patients (19.5%) (Table 3). No diagnosis-specific epileptiform pattern was identified. Clinical video recordings, when available, were systematically reviewed and used as supportive diagnostic tools to differentiate epileptic from non-epileptic events (Supplementary Videos S1–S9).

Importantly, no patients were observed to develop epilepsy during the follow-up period, and all patients were clinically followed. The majority of patients demonstrated spontaneous clinical improvement, consistent with the benign nature of DBMD.

## 4. Discussion

In this retrospective cohort study, developmental and benign movement disorders (DBMD) accounted for nearly one in four children referred with suspected seizures, highlighting the substantial contribution of non-epileptic conditions to pediatric seizure referrals. This finding underscores the critical importance of accurate differential diagnosis in clinical practice and suggests that a significant proportion of children may be exposed to unnecessary diagnostic procedures or treatments [7,8,9].

The predominance of breath-holding spells in our cohort is consistent with previous epidemiological studies reporting incidence rates ranging from 4% to 27% in otherwise healthy infants [4,5]. These events are widely considered manifestations of autonomic dysregulation and exaggerated vagal responses during early neurodevelopment [4,5,6]. The early age clustering observed in our data further supports the hypothesis that immature autonomic control mechanisms play a central role in their pathophysiology.

Sandifer syndrome and non-epileptic staring episodes represent important diagnostic challenges due to their close clinical resemblance to epileptic seizures. As previously reported, Sandifer syndrome is frequently misinterpreted as focal epilepsy, leading to unnecessary neurological investigations and treatment [10]. Similarly, non-epileptic staring episodes are often confused with absence seizures despite normal electroencephalographic findings [9,10,11]. These overlaps highlight the complexity of distinguishing epileptic from non-epileptic paroxysmal events in pediatric populations.

A key finding of this study is the clear age-dependent distribution of DBMD subtypes. Consistent with previous literature, many benign paroxysmal movement disorders are thought to reflect transient immaturity of neuronal networks rather than epileptogenic processes [1,2]. The earlier onset of breath-holding spells, intermediate presentation of Sandifer syndrome, and later occurrence of staring episodes suggest a developmental continuum related to maturation of autonomic, motor, and attentional systems [11,12,13,14]. This pattern supports a neurodevelopmental framework in which DBMD represent physiological expressions of evolving cortical–subcortical connectivity.

Misdiagnosis of epilepsy remains a major clinical concern. Previous studies have shown that 20–30% of children initially diagnosed with epilepsy are ultimately found to have non-epileptic conditions [7,9,12]. In this context, our finding that no patients were observed to develop epilepsy during the follow-up period provides strong evidence supporting the benign and non-epileptic nature of DBMD. However, although the follow-up duration was substantial, late-onset epilepsy cannot be entirely excluded. This observation emphasizes the importance of avoiding premature epilepsy diagnosis and unnecessary antiepileptic treatment.

The predominance of normal EEG findings in our cohort (80.5%) is consistent with prior studies demonstrating that epileptiform abnormalities are uncommon in benign non-epileptic paroxysmal disorders [3,8,13]. Non-specific EEG abnormalities may contribute to diagnostic uncertainty when interpreted without appropriate clinical correlation, potentially leading to misdiagnosis [13,14,15,16]. These findings reinforce the need for careful integration of clinical history, video documentation, and developmental context in diagnostic decision-making.

The relatively high prevalence observed in this study may be influenced by referral bias, as the study was conducted in a tertiary care center where more complex cases are typically evaluated. In addition, the moderate statistical power of the study may limit the ability to detect smaller differences between diagnostic subgroups.

Motor stereotypies and other non-epileptic movement phenomena observed in our cohort are well-recognized benign developmental behaviors in typically developing children [9,17]. Taken together, our results highlight the broad spectrum of physiological and maturational motor phenomena that may mimic epileptic seizures.

In clinical practice, recognition of age-specific patterns and predominantly normal EEG findings may help clinicians avoid unnecessary investigations and provide appropriate reassurance to families. Importantly, this study adds to the existing literature by providing data from a relatively large cohort of children referred specifically for suspected seizures, thereby offering clinically relevant insights into real-world diagnostic challenges. The identification of distinct age-related patterns may assist clinicians in differentiating DBMD from epilepsy and improving diagnostic accuracy in routine practice.

### Limitations

This study has several limitations. Its retrospective single-center design may limit generalizability, and referral bias is possible, as tertiary centers often evaluate more complex cases. Prolonged video-EEG monitoring was not systematically performed in all patients; therefore, subtle epileptic events cannot be entirely excluded. In addition, EEG recordings during clinical events were not consistently available.

In addition, the moderate statistical power of the study may limit the ability to detect smaller differences between diagnostic subgroups. However, the consistent clinical findings and absence of epileptic evolution during follow-up support the reliability of the diagnoses.

Standardized prospective video-analysis protocols were not applied. Nevertheless, the relatively large sample size and the combined use of clinical assessment, EEG findings, and video recordings strengthen the overall validity of the study. Although all patients were clinically followed, the follow-up duration, while substantial, may not completely exclude the possibility of late-onset epilepsy. Further multicenter prospective studies are warranted to confirm these findings.

## 5. Conclusions

Developmental and benign movement disorders represent a substantial and clinically significant proportion of children referred with suspected seizures. Their distinct age-related presentation patterns, predominantly normal EEG findings, and favorable clinical outcomes strongly support a neurodevelopmental rather than epileptic origin. Improved recognition of these conditions is essential to reduce epilepsy misdiagnosis, avoid unnecessary antiepileptic treatment, and minimize the associated healthcare burden. Integrating developmental context, detailed clinical assessment, and cautious interpretation of EEG findings into routine practice may significantly enhance diagnostic accuracy and optimize pediatric neurological care.

## Figures and Tables

**Table 1:** Demographic characteristics of patients with developmental and benign movement disorders (DBMD).

Variable	Value	Statistical Test	Statistic	*p*-Value	Effect Size
Female, n (%)	62 (54.9)	χ^2^ test	χ^2^ = 7.02	0.135	Cramer’s V = 0.18
Male, n (%)	51 (45.1)	–	–	–	–
Age at presentation (months), mean ± SD	14.2 ± 9.6	One-way ANOVA	F = 5.12	0.001	Cohen’s f = 0.27
Follow-up duration (months), mean ± SD	44.4 ± 18.5	–	–	–	–

Values are presented as mean ± standard deviation (SD) or number (percentage), as appropriate. The association between sex and diagnostic subgroups was analyzed using the chi-square test. Age differences among diagnostic groups were assessed using one-way ANOVA with Tukey post hoc analysis. Effect sizes are reported as Cramer’s V for chi-square test and Cohen’s f for ANOVA. A *p*-value < 0.05 was considered statistically significant.

**Table 2:** Diagnostic distribution of developmental and benign movement disorders (DBMDs).

Diagnosis	n	% (Total Cohort, n = 453)	% (DBMD Group, n = 113)
Breath-holding spells	30	6.6	26.5
Sandifer syndrome	15	3.3	13.3
Non-epileptic staring episodes	12	2.6	10.6
Benign sleep myoclonus	10	2.2	8.8
Shuddering attacks	8	1.8	7.1
Motor stereotypies	8	1.8	7.1
Non-epileptic myoclonus/gratification disorder	5	1.1	4.4
Tremor and other rare diagnoses	25	5.5	22.1
Total DBMDs	113	24.9	100

Percentages are calculated relative to both the total screened cohort (n = 453) and the DBMD subgroup (n = 113).

**Table 3:** Electroencephalographic (EEG) findings in patients with developmental and benign movement disorders (DBMDs).

EEG Finding	n	% (n = 113)
Normal	91	80.5
Non-specific abnormalities *	22	19.5
Epileptiform activity	0	0

* Non-specific abnormalities included intermittent slowing, background irregularity, and age-inappropriate sharp transients without epileptiform morphology.

## Data Availability

The datasets generated and/or analyzed during the current study are available from the corresponding author on reasonable request.

## Supplementary Materials

The following supporting information can be downloaded at: https://www.mdpi.com/article/10.3390/children13050650/s1. Supplementary Video S1: Downward gaze deviation; Supplementary Video S2: Head shaking episodes; Supplementary Video S3: Benign torticollis; Supplementary Video S4: Benign myoclonus of early infancy; Supplementary Video S5: Sandifer syndrome; Supplementary Video S6: Shuddering attacks; Supplementary Video S7: Stereotypic movements; Supplementary Video S8: Transient dystonia of infancy; Supplementary Video S9: Upward tonic gaze. Supplementary video files are provided to illustrate representative clinical examples of developmental and benign movement disorders observed in this cohort. All video recordings were obtained during routine clinical evaluation with written informed parental consent. All data were anonymized prior to submission to ensure patient confidentiality.
