# Supplementary material for: Benign Movement Disorders Mimicking Seizures in Children: A Retrospective Cohort Study
Source: Children (Basel). 2026 May 6;13(5):650. doi: 10.3390/children13050650 (PMC13204290; doi:10.3390/children13050650)
Supplement: Supplementary file 1 [file children-13-00650-s001.zip › children-4277238-supplementary.pdf]

Supplementary Video Material

The following video recordings are provided as supplementary material to illustrate representative clinical examples of developmental and benign movement disorders observed in our cohort:

- **Supplementary Video S1:** Downward gaze deviation
- **Supplementary Video S2:** Head shaking episodes
- **Supplementary Video S3:** Benign torticollis
- **Supplementary Video S4:** Benign myoclonus of early infancy
- **Supplementary Video S5:** Benign sleep myoclonus
- **Supplementary Video S6:** Sandifer syndrome
- **Supplementary Video S7:** Shuddering attacks
- **Supplementary Video S8:** Stereotypic movements
- **Supplementary Video S9:** Transient dystonia of infancy
- **Supplementary Video S10:** Upward tonic gaze

All videos were recorded during routine clinical evaluation with written informed parental consent. Patient identifiers have been removed to ensure confidentiality.
